# Coenzyme Q10 Ameliorates Pain and Cartilage Degradation in a Rat Model of Osteoarthritis by Regulating Nitric Oxide and Inflammatory Cytokines

**DOI:** 10.1371/journal.pone.0069362

**Published:** 2013-07-22

**Authors:** Jennifer Lee, Yeon Sik Hong, Jeong Hee Jeong, Eun Ji Yang, Joo Yeon Jhun, Mi Kyoung Park, Young Ok Jung, Jun Ki Min, Ho Youn Kim, Sung Hwan Park, Mi-La Cho

**Affiliations:** 1 Division of Rheumatology Department of Internal Medicine The Catholic University of Korea, Seoul, Korea; 2 Rheumatism Research Center, College of Medicine, The Catholic University of Korea, Seoul, Korea; 3 Division of Rheumatology, Department of Internal Medicine, Hallym University Kang Nam Sacred Heart Hospital, Seoul, Korea; 4 Conversant Research Consortium in Immunologic disease, College of Medicine, The Catholic University of Korea, Seoul, Korea; University of Szeged, Hungary

## Abstract

**Objective:**

To investigate the effect of CoenzymeQ10 (CoQ10) on pain severity and cartilage degeneration in an experimental model of rat osteoarthritis (OA).

**Materials and Methods:**

OA was induced in rats by intra-articular injection of monosodium iodoacetate (MIA) to the knee. Oral administration of CoQ10 was initiated on day 4 after MIA injection. Pain severity was assessed by measuring secondary tactile allodynia using the von Frey assessment test. The degree of cartilage degradation was determined by measuring cartilage thickness and the amount of proteoglycan. The mankin scoring system was also used. Expressions of matrix metalloproteinase-13 (MMP-13), interleukin-1β (IL-1β), IL-6, IL-15, inducible nitric oxide synthase (iNOS), nitrotyrosine and receptor for advanced glycation end products (RAGE) were analyzed using immunohistochemistry.

**Results:**

Treatment with CoQ10 demonstrated an antinociceptive effect in the OA animal model. The reduction in secondary tactile allodynia was shown by an increased pain withdrawal latency and pain withdrawal threshold. CoQ10 also attenuated cartilage degeneration in the osteoarthritic joints. MMP-13, IL-1β, IL-6, IL-15, iNOS, nitrotyrosine and RAGE expressions were upregulated in OA joints and significantly reduced with CoQ10 treatment.

**Conclusion:**

CoQ10 exerts a therapeutic effect on OA via pain suppression and cartilage degeneration by inhibiting inflammatory mediators, which play a vital role in OA pathogenesis.

## Introduction

Osteoarthritis (OA) has been defined as a degenerative disease involving an increased pressure on a particular joint or a degeneration of cartilage matrix, resulting in a loss of cartilage. However, the current paradigm of OA has shifted from the concept of “wear and tear” disease to the inflammation-mediated disease [1]. Inflammatory mediators such as cytokines, chemokines and reactive oxygen species (ROS) are produced in OA joint tissues (synovium, cartilage and subchondral bone), which ultimately affect joint tissues leading to the release of matrix metalloproteinases (MMPs) and eventually cartilage degradation [2]. Although OA is the most common joint disease causing functional disability, disease modifying OA drugs (DMOAD) are still lacking, and current treatments mainly focus on pain relief. Recent advances in understanding the pathogenesis OA is expected to lead to better therapies that can modify the disease progression.

Coenzyme Q10 (CoQ10), also known as ubiquinone-10, is a lipid with a structure consisting of 1,4-benzoquinone and side chain of 10 isoprenyl subunits. The essential role of CoQ10 is to produce adenosine triphosphate (ATP) in the mitochondria as a coenzyme for mitochondrial enzymes, which are involved in oxidative phosphorylation pathway [3]. Additionally, CoQ10 is known to be a powerful antioxidant that can inhibit peroxidation of the cell membrane lipids and plasma lipoproteins, thus preventing atherosclerosis [4]. More recently, several studies have also shown the anti-inflammatory effects of CoQ10 [5]–[7], and the therapeutic role of CoQ10 in inflammatory disorder has been investigated. Buerova *et al.* showed that treatment with CoQ10 had an antiarthritic (decrease of hind paw volume) and antioxidative effect in adjuvant induced arthritis model [8]. As OA is regarded as a disease of perpetuating low grade inflammation, it is plausible that CoQ10 might have a therapeutic role in OA as well. To our knowledge, a therapeutic effect of CoQ10 in an OA animal model has never been published. In this study, the effect of CoQ10 on pain and cartilage degradation in a rat model of OA was investigated.

## Materials and Methods

### Animals

Male Wistar rats weighing 140–230 g (6 weeks of age) at the start of the experiment were purchased from Central Lab. Animal Inc. (Seoul, South Korea). The animals were housed three per cage in a room with controlled temperature conditions (21–22°C) with controlled lighting (12-h light/12-h dark cycle) and had access to sterile food and water. All animal procedures were approved by the Animal Research Ethics Committee at the Catholic University of Korea.

### Induction of OA in the Rat and Treatment with Coenzyme Q10

The animals were randomized and assigned to treatment groups prior to the start of the study. After anesthetization with isoflurane, rats were injected with 50µl containing 3 mg of MIA (Sigma) using a 26.5-G needle inserted through the patellar ligament into the intra-articular space of the right knee. Control rats were injected with an equivalent volume of saline. CoQ10 was kindly provided by Daewoong Pharmaceutical Company (Seoul, Korea). CoQ10 dissolved in cotton seed oil was administered orally every day. The vehicle-treated animals were given an equivalent volume of cotton seed oil solution. The dose of CoQ10 was 100 mg/kg.

### Assessment of Pain Behavior

The MIA-treated rats were randomized to each experimental group. The nociceptive testing was performed using a dynamic plantar esthesiometer (Ugo Basile), an automated version of the von Frey hair assessment procedure, before the MIA injection (Day 0) and on the given day after MIA injection. The rats were placed on a metal mesh surface in an acrylic chamber in a temperature-controlled room (21–22°C) and allowed to rest for 15 min before testing. The touch stimulator unit was oriented beneath the animal. An adjustable angled mirror was used to place the stimulating microfilament (0.5-mm diameter) below the plantar surface of the hind paw. When the instrument was activated, a fine plastic monofilament advanced at a constant speed and touched the paw in the proximal metatarsal region. The filament exerted a gradual increasing force on the plantar surface, starting below the threshold of detection and increasing until the stimulus became painful indicated by the removal of its paw. The force required to elicit a paw withdrawal reflex was recorded automatically and measured in g. A maximum force of 50 g and a ramp speed of 20 s were used for all esthesiometry tests. Pain behavioral tests of secondary tactile allodynia were conducted right before the CoQ10 administration.

### Histological and Immunohistochemical Analyses

Histological changes were assessed to confirm the effect of CoQ10 on cartilage degeneration in the knee joints of OA rats. The animals were perfused via the ascending aorta with 10% neutral buffered formalin (pH 7.4). The knee joints, including the patella and joint capsule, were resected and kept in the same fixative for an additional 48 h at 4°C. The fixed specimens were decalcified with 5% formic acid decalcifier for 6 days at 4°C. After decalcification, the specimens were embedded in parafin. Standardized 7-mm serial sections were obtained at the medial and lateral midcondylar level in the sagittal plane and were stained with hematoxylin and eosin (HE), Safranin O-fast green, and toluidine blue to enable evaluation of proteoglycan content. Slides for immunohistochemistry were deparafinized and rehydrated using a graded ethanol series. The sections were depleted of endogenous peroxidase activity by adding methanolic H2O2 and then blocked with normal goat serum for 30 min. The samples were incubated overnight at 4°C with antibodies to IL-1β at a dilution of 1∶50 (Santa Cruz Biotechnology, Santa Cruz, CA, USA), matrixmetalloproteinase-13 (MMP-13) at 1∶50 (Abcam, Cambridge, UK), IL-6 at 1∶50 (Abcam), IL-15 at 1∶50 (Santa Cruz), inducible nitric oxide synthase (iNOS) at 1∶100 (Abcam), nitrotyrosine 1∶100 (Santa Cruz) or receptor advanced glycation end product (RAGE) at 1∶50 (Santa Cruz). The samples were then incubated with the respective secondary antibodies, biotinylated anti-mouse IgG or rabbit IgG, for 20 min, conjugated to a streptavidine peroxidase complex (Vector Laboratories, Burlingame, CA, USA) for 1 h, and finally with 3,30-diaminobenzidine (Dako, Glostrup, Denmark). The sections were counterstained with Mayer’s hematoxylin and photographed using an Olympus photomicroscope (Olympus, Tokyo, Japan).

A modified Mankin’s histological score [9] (original score proposed by Mankin et al. [10]) was used to score histological injuries of the articular cartilage as follows. The structure was scored on a scale of 0–6, where 0 = normal; 1 = irregular surface, including fissures into the radial layer; 2 = pannus; 3 = absence of superficial cartilage layers; 4 = slight disorganization (cellular row absent, some small superficial clusters); 5 = fissure into the calcified cartilage layer; and 6 = disorganization (chaotic structure, clusters, and osteoclasts activity). Cellular abnormalities were scored on a scale of 0–3, where 0 = normal; 1 = hypercellularity, including small superficial clusters; 2 = clusters; and 3 = hypocellularity. The matrix staining was scored on a scale of 0–4, where 0 = normal/slight reduction in staining; 1 = staining reduced in the radial layer; 2 = staining reduced in the interterritorial matrix; 3 = staining present only in the pericellular matrix; and 4 = staining absent. Joint space width was estimated by sum of the nearest distance of medial and lateral tibiofemoral joints. Histological evaluation was performed by two independent experienced researchers who were blinded to treatment arm.

### Statistical Analysis

The change of pain behavior is expressed as means ± standard error of the mean (S.E.M.). Each value of histological assessments and pain behaviors was represented as a dot plot. One-way analysis of variance followed by Bonferroni’s post-hoc test was used to compare pain and histological scores. To assess the Gaussian distribution and the equality of variance, Shapiro-Wilk test and Levene’s test were used, respectively. The program used for the statistical analysis was SPSS statistical software package standard version 16.0 (SPSS Inc., Chicago, IL, USA). P values less than 0.05 (two-tailed) were considered significant.

## Results

### Coenzyme Q10 Exerted Antinociceptive Effect on MIA-induced OA Rats

**Figure 1:**
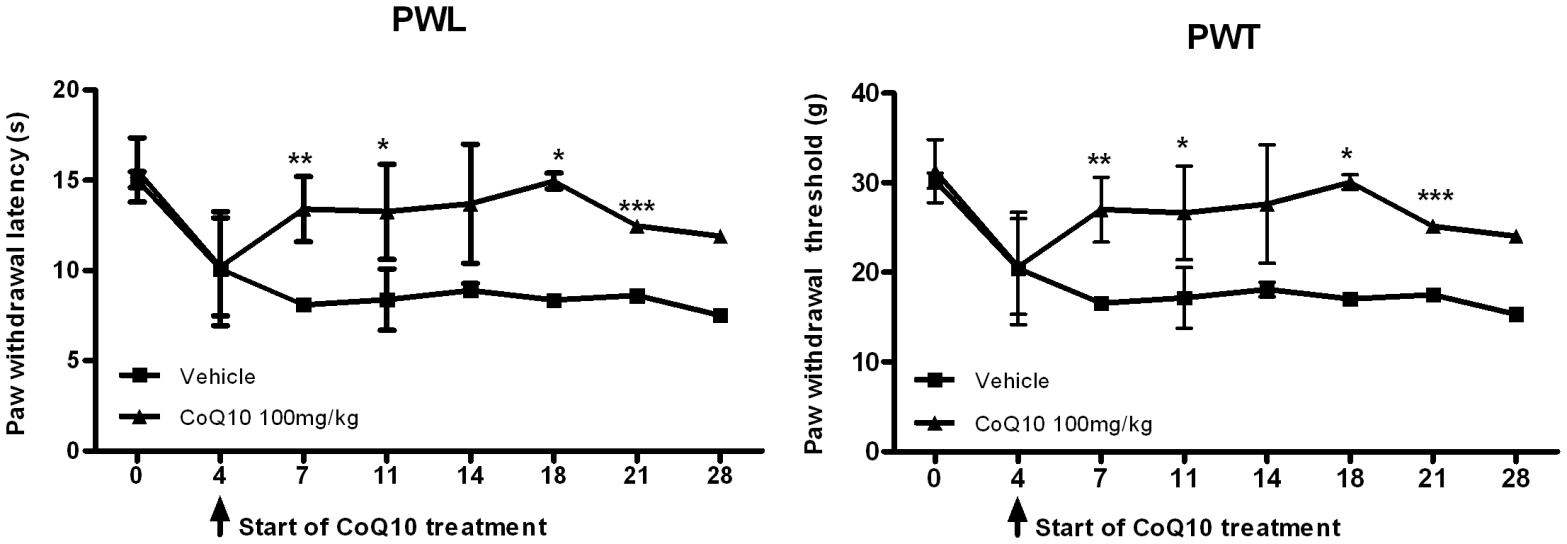
Therapeutic effect of Coenzyme Q10 in an early phase of MIA-induced OA in rats. Rats were injected with 3 mg of monosodium iodoacetate (MIA) in the right knee. Coenzyme Q10 (CoQ10) was administered orally every day from day 4 after MIA injection. Behavioral tests of secondary tactile allodynia in MIA-injected rats treated with vehicle or CoQ10 were evaluated using a dynamic plantar esthesiometer (n = 10 on each day for each group). Compared with vehicle-treated OA rats, OA animals treated with CoQ10 at a dose of 100 mg/kg showed a significant increase in PWL and PWT. The data are expressed as mean and error bars for three animals per group. PWL and PWT were conducted right before the administration of CoQ10. Significant differences between vehicle- and CoQ10-treated groups: *P<0.05, **P<0.01 and ***P<0.001 compared with the vehicle-treated OA group.

To evaluate the ability of CoQ10 to reduce the pain in OA, the secondary tactile allodynia in MIA-induced OA rats was assessed. In automated von Frey hair assessment tests, the paw withdrawal latency (PWL) and the paw withdrawal threshold (PWT) were increased in the group of OA rats treated with CoQ10 (figure 1), indicating that CoQ10 exerted antinociceptive effects.

### Cartilage Damage was Abrogated with the Treatment of Coenzyme Q10 in OA Rats

**Figure 2:**
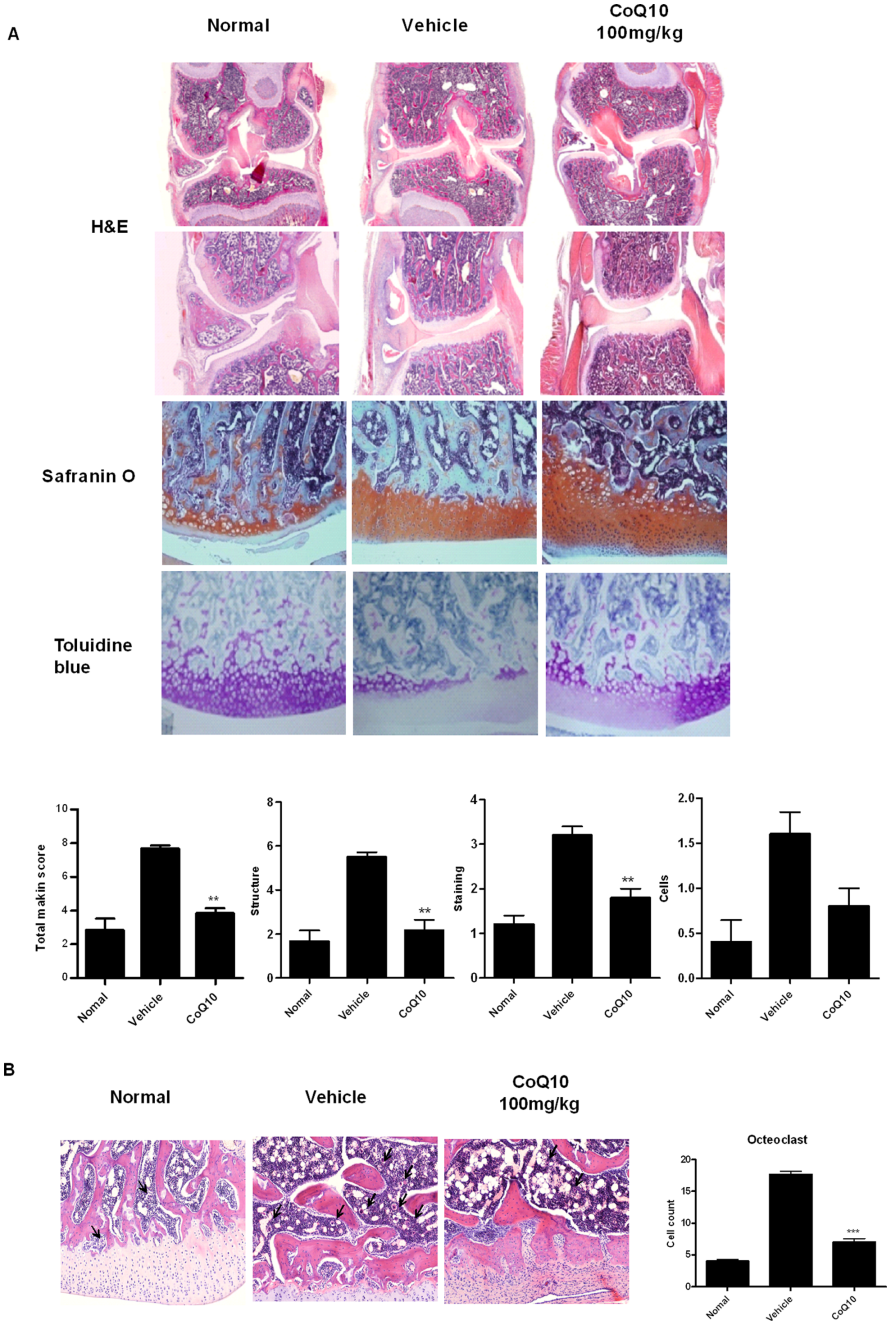
Histological evaluation of joints and osteoclastic activity after treatment with CoenzymeQ10 in MIA-induced OA. Rats were injected with 3 mg of monosodium iodoacetate (MIA) in the right knee. Coenzyme Q10 (CoQ10) was administered orally every day from the day 4 after MIA injection. The knee joints were resected on day 7 after MIA injection. (A) The knee joints from the OA rats treated with CoQ10 or vehicle were stained with HE, Safranin O-fast green, and toluidine blue. Extensive cartilage degradation, bone destruction, and fibrosis are seen in the vehicle-treated group, whereas the treatment with CoQ10 preserved the articular space and prevented the depletion of proteoglycan. CoQ10 treatment preserved the cartilage structure and decreased the depth and the extent of cartilage damage. The joint lesions were graded on a scale of 0–13 using the modified Mankin scoring system. Total mankin score is a sum of the scores for cartilage structure, cellular abnormalities, and matrix staining. CoQ10 prevented damage to the cartilage structure, reduced cellular abnormalities, and preserved matrix staining. The data are expressed as mean and error bars for six animals per group. (B) The number of osteoclasts were measured in the knee joint sections. Osteoclasts were more frequently found in vehicle-treated group than CoQ10-treated group. *P<0.05, **P<0.01, and ***P<0.001 compared with the MIA-injected vehicle-treated group.

As the cartilage degradation is the main characteristic of OA joints, the improvement of cartilage degradation of OA joints with CoQ10 treatment was investigated. On day 7 after MIA injection, the knees were resected and the cartilages were stained with HE, Safrainin O-fast green, and toluidine blue. The cartilage thickness and the content of proteoglycan in joints of OA rats were reduced compared to healthy controls. In the CoQ10 treated group, cartilage degradation was prevented until 28 days after MIA injection (figure 2a). The degree of cartilage degradation was also assessed by Mankin’s score system, which consists of scores on structural damage, cellular abnormalities, and matrix staining. The CoQ10 treated group also showed a significantly lower Mankin score than the vehicle treated group (figure 2a). The number of osteoclasts were increased after MIA injection and reduced with CoQ10 treatment (figure 2b).

### The Expression of Matrix Metalloproteinase-13 (MMP-13), IL-1β, IL-6 and IL-15 Decreased with Treatment of Coenzyme Q10 in OA Rats

**Figure 3:**
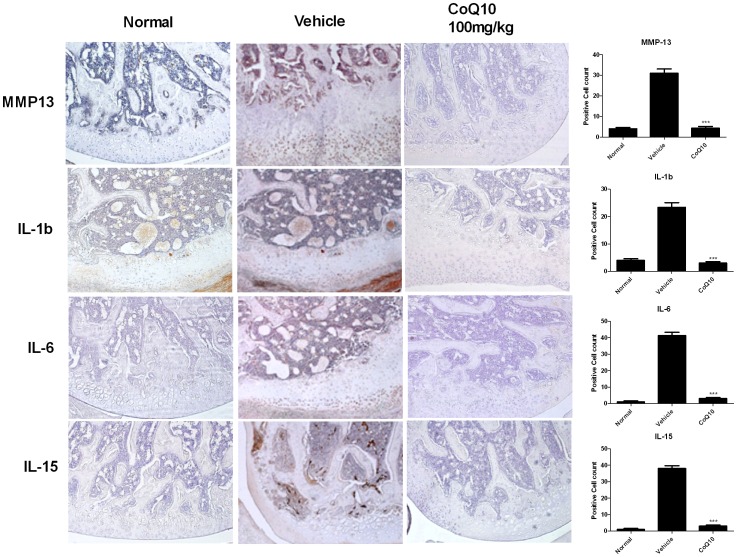
Effect of Coenzyme Q10 on the expression of MMP-13, IL-1β, IL-6 and IL-15 in OA joints. Rats were injected with 3 mg of MIA in the right knee. Coenzyme Q10 was administered orally every day from the day 4 after MIA injection. Immunohistochemical staining was used to identify the expression of MMP-13, IL-1b, IL-6, and IL-15 in the articular cartilage. In the articular cartilage of MIA-injected rats, the expression of MMP-13, IL-1β, IL-6 and IL-15 increased, whereas treatment with CoQ10 inhibited the expression of these molecules. The data are expressed as mean and error bars for six animals per group. Original magnification 200x. *P<0.05, **P<0.01, and ***P<0.001 compared with the MIA-injected vehicle-treated group.

To elucidate the mechanism of chondroprotective effect of CoQ10, the expression of matrix degrading enzyme matrix metalloproteinase-13 (MMP-13) was measured. MMP-13 expression increased after MIA injection, whereas treatment with CoQ10 abrogated this increase (figure 3). Furthermore, proinflammatory cytokines, IL-1β, IL-6, and IL-15 expressions were also determined. Results showed that these cytokines were upregulated in the OA joints and failed to maintain high expression with CoQ10 treatment (figure 3).

### The Expression of Inducible Nitric Oxide Synthase (iNOS) and Receptor for Advanced Glycation End Products (RAGE) Expression was Diminished with the Treatment of Coenzyme Q10 in OA Rats

**Figure 4:**
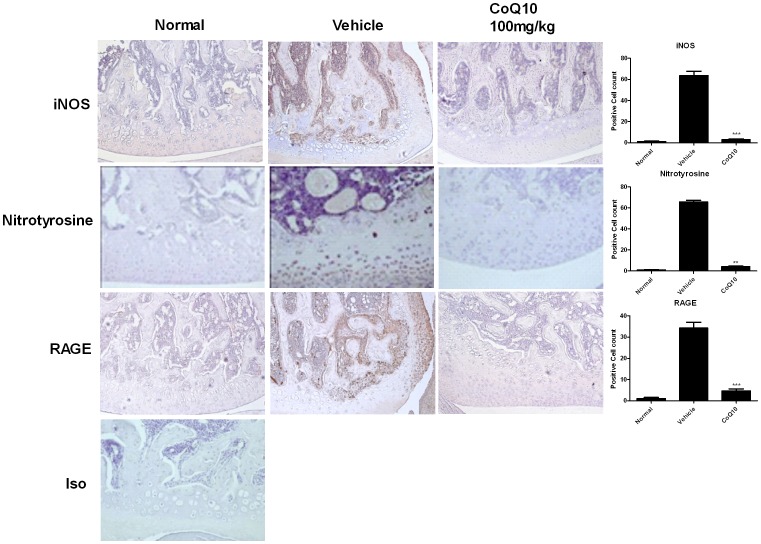
Attenuated iNOS, nitrotyrosine and RAGE expression in Coenzyme Q10-treated OA rats. Rats were injected with 3 mg of monosodium iodoacetate (MIA) in the right knee. Coenzyme Q10 (CoQ10) was administered orally every day from the day 4 after MIA injection. The knees were resected on day 7 after MIA injection. Immunohistochemical staining was used to identify the expression of induced nitric oxide synthase (iNOS), nitrotyrosine and receptor for advanced glycation end products (RAGE) in the joint tissue. Expression of iNOS, nitrotyrosine and RAGE in chondrocytes was increased after MIA-injection, and was reduced in CoQ10-treated OA animals. The data are expressed as mean and error bar for three animals per group. Original magnification 200x. *P<0.05, **P<0.01, and ***P<0.001 compared with the MIA-injected vehicle-treated group.

As nitric oxide (NO) [11] and receptor for advanced glycation end products (RAGE) [12], [13] have been implicated in the OA pathogenesis, the expression of inducible NO synthase (iNOS), nitrotyrosine and RAGE in OA joints were determined using immunohistochemistry. The expression of iNOS, nitrotyrosine and RAGE were increased in OA joints (figure 4). Treatment with CoQ10 from day 4 after MIA injection abrogated the elevated expression (figure 4).

## Discussion

The present study demonstrated the therapeutic effect of CoQ10 in an OA animal model for the first time. Treatment with CoQ10 ameliorated arthritic pain and reduced the cartilage damage.

The mechanism underlying OA-related pain has not been fully understood. As articular cartilage is avascular and aneural, non-cartilagenous joint tissues including subchondral bone, periosteum, synovium, ligament, and the joint capsules are thought to be the sources of pain generation [14]. Pain-generating proinflammatory cytokines including IL-1β and IL-6 [2] and the effect of NO derivative on subchondral bone [15] have been suggested to be essential in OA pain. Our result showed that the expression of IL-1β, IL-6 and iNOS were reduced with CoQ10 treatment, potentially explaining the reduced secondary tactile allodynia measured by von Frey hair test in our model. With the lack of direct and standardized test which can reflect the pain severity in OA animal model, von Frey hair test has been used as a pain-estimating procedure. Nevertheless, as it cannot distinguish between pain and simple mechanically annoying stimuli, the results should be interpreted prudently. In our study, the reduced secondary tactile allodynia was accompanied by decreased expression of pain-generating cytokines in the arthritic joints, supporting that the results of Frey test reflected the pain severity.

The antinociceptive effect of CoQ10 has been widely accepted in treating statin-induced myalgia. Recently, it was also shown to be effective in headache of fibromyalgia patients [16]. The authors argued that the pain in these diseases was associated with mitochondrial dysfunction, which could be restored by CoQ10 supplement. Provided that mitochondrial dysfunction has also been implicated in OA pathogenesis [17]–[19], CoQ10 may reduce the OA pain through a similar mechanism.

MIA, an inhibitor of glyceraldehyde-3-phosphate dehydrogenase, was used to induce OA like lesion in rat joints as it causes cartilage degradation resembling the pathological changes of human OA. Similar to the implication of ROS and mitochondrial dysfunction in human OA, MIA-induced chondrocyte apoptosis was recently reported to be mediated by the mitochondrial pathway involving ROS production and caspase activation [20]. This findings further support that the MIA model can represent human OA as it shows similar mechanism of cartilage degradation. In our rat OA model, CoQ10 appears to have abrogated MIA-induced mitochondrial dysfunction and contributed to pain reduction.

It is now believed that sustained low grade inflammation plays a key role in OA pathogenesis [21]. We demonstrated that the expression of IL-1b, IL-6 and IL-15 increased in the OA cartilage. IL-1β is known to reduce type II collagen expression in chondrocytes [22], increase MMP [23], and induce iNOS expression [2]. IL-6 was reported to be elevated in knee OA joints [24]. In concert with IL-1β, IL-6 upregulated MMP-1 and MMP-13 expression in human cartilage extract culture [25]. IL-15, an inflammatory cytokine associated with the recruitment and survival of CD8+ T cells, is known to be elevated in the joints of early OA and involved in MMP-1, -3 expression [26]. The reduction seen in these cytokines after CoQ10 treatment further supports the therapeutic potential of CoQ10 in OA.

RAGE belongs to the immunoglobulin superfamily, was firstly described as a receptor for advanced glycation end products (AGE) [27] which are non-enzymatically glycosylated proteins or lipids that develop in normal aging or inflammatory process, especially in diabetes. RAGE has emerged as a pattern recognition receptor. To date, a diverse repertoire of ligands including S100A [28] and the high mobility group box chromosomal protein 1(HMGB-1) [29] were identified. The ligation of RAGE induces multiple signaling pathways resulting in the activation of several transcription factors such as NF-kB, which, in turn, increases proinflammatory cytokines [30]. In OA, RAGE has been reported to be increased in OA tissues [31]. Ligation of RAGE induced production of proinflammatory cytokines such as IL-6, IL-8 [32], and TNF-α as well as increasing MMP-1,-3,-13 in OA joints [33]. As AGE accumulates in articular cartilage with aging, implication of RAGE in OA can be a good explanation for the most relevant risk factor for OA – aging [34]. Consistent with these findings, our results showed that the expression of RAGE increased in the joints of the OA model compared to the healthy control. Moreover, the reduced RAGE expression after CoQ10 treatment may explain the decreased expression of IL-6 and MMP-13.

Tsai *et al.* reported that CoQ10 suppresses iNOS and protected endothelial cells from oxidative stress-induced injury [35]. As well, our result showed that the expression of iNOS was reduced after CoQ10 treatment in OA joints. The increased iNOS expression reflects the increased NO in the OA joints. The role of NO in the pathogenesis of OA has been recently described in detail [11]. The most renounced role of NO is mediated by peroxynitrite, which is generated by combination of NO and ROS. Peroxynitrite induces tissue oxidative damage and apoptosis of chondrocytes. Moreover, NO itself is associated with subchondral bone sclerosis, which has emerged as critical in OA pathogenesis in terms of bone remodeling [36]. Proinflammatory cytokine such as IL-1β induces NO, thus activating NF-kB and producing proinflammatory cytokine. Therefore, the ability of CoQ10 to break this cycle by suppressing iNOS could contribute to OA treatment.

In addition, the association of adipokine and inflammation and OA was recently reported [37]. Patients with metabolic syndrome were reported more likely to develop OA than people with simple obesity [38], where administration of CoQ10 attenuated the increase of oxidative and nitrative stress markers and inflammatory markers in a rat model of metabolic syndrome [6]. As mitochondrial dysfunction has been reported in metabolic syndrome [39]–[42], the therapeutic effect of CoQ10 appears to be related to the restoration of mitochondrial function, at least in part. All together, CoQ10 may be a good therapeutic option for OA targeting various pathogenetic mechanisms of the disease including inflammation, dysregulated metabolism, oxidative stress, and mitochondrial dysfunction.

In conclusion, this is the first study addressing the therapeutic potential of the CoQ10 in the OA animal model. CoQ10 reduced the pain, ameliorated hisotologic score of the paw with arthritis, and diminished oxidative stress in the OA animal model. Considering the bioenergetic, antioxidant and anti-inflammatory property of CoQ10, this may be a promising therapeutic option for OA, a consequence of inflammation and dysregulated metabolism.
